# 1120. Case Study. Antifungal and Surgical Approach in a Pediatric Burn Patient with Multiple Drug-resistant Fungi

**DOI:** 10.1093/jbcr/irag033.423

**Published:** 2026-04-06

**Authors:** Laura Steffens, Colton Radford, Denis Vanini, Callie M Thompson, Giavonni Mystique Lewis, Irma D Fleming, Christopher R LaChapelle

**Affiliations:** University of Utah Health Burn Center, Salt Lake City, UT; University of Utah Health Burn Center, Salt Lake City, UT; University of Utah Health Burn Center, Salt Lake City, UT; University of Utah Health, Salt Lake City, UT; University of Utah Health, Salt Lake City, UT; University of Utah Health Burn Center, Salt Lake City, UT; University of Utah Health, Salt Lake City, UT

## Abstract

**Patient Presentation (age range, injury details, relevant history):**

This is a case of a 9-year-old, 53 kg male patient who sustained 50% total body surface area burns secondary to a high-speed vehicle crash and prolonged extrication. After several debridements at an outside hospital, he was transferred to our facility for amputations, further surgical intervention, and renal replacement therapy.

**Clinical Challenges:**

Throughout the patient’s stay, he notably grew multidrug-resistant fungi from burn wounds and surgical sites. Literature guidance is sparse related to this patient’s unique characteristics, particularly with regard to aggressive dosing of antifungals to treat high MIC organisms in a hyperdynamic, pediatric patient. Data regarding synergy of antifungals in the treatment of various drug-resistant fungi is also lacking. As fungal infections account for 20-25% of burn infections and portend high morbidity and mortality, early source control in conjunction with effective antimicrobial agents is imperative.

**Management Approach:**

On arrival, the patient underwent bilateral guillotine below-knee amputations for 4th degree burn injuries. He subsequently underwent a lengthy, staged series of surgeries to treat his burn wounds and address infections, accompanied by multiple courses of antimicrobials for bacterial and fungal infections. He notably grew Nakaseomyces glabrata (caspofungin 0.12) from his right leg post-excision cultures and caspofungin 70 mg/mg2/day was initiated and continued for a 35-day course. Fusarium species (amphotericin 4, caspofungin >16, voriconazole >16, posaconazole >16), Nakaseomyces glabrata, and Actinomucor elegans (amphotericin 1, posaconazole 0.25, voriconazole 4) grew on hospital day 8. Voriconazole was initiated at 9 mg/kg every 12 hours for two doses, followed by 8 mg/kg every 12 hours. Doses were escalated over 2 weeks to maintenance dose of 10 mg/kg every 12 hours to achieve a trough level of 1.1 μg/mL. Systemic liposomal amphotericin was added on day 10, initially dosed at 5 mg/kg/day until speciation was known, and escalated to 10 mg/kg/day with resulting susceptibilities of Actinomucor and Fusarium species. Voriconazole and amphotericin were maintained for synergy during active infection. At day 39, voriconazole was stopped and transitioned to posaconazole for a 48-day treatment course for Actinomucor. Amphotericin was continued for a total 43-day course for Fusarium treatment.

**Outcomes:**

The patient was discharged to a pediatric rehabilitation facility on day 116. He experienced no adverse effects that were attributed to his antifungal therapy.

**Lessons Learned:**

The outcomes noted in this case report were attributed to aggressive source control, in combination with antifungal agents for the treatment of invasive fungal infections. In the setting of a large burn in a hyperdynamic pediatric patient, we found it would be beneficial to be even more aggressive with dosing of antifungals up front as the patient tolerated it well with no adverse effects and survived to discharge.

**Applicability to Practice:**

This case report adds to the growing body of literature surrounding antifungal use alongside surgical intervention for drug-resistant fungal organisms, pediatric, and hyperdynamic burn patients. It also highlights the importance of combination therapy up front to harness synergistic effects, as well as empirically cover slower growing organisms.

